# Using the Optical Fractionator to Estimate Total Cell Numbers in the Normal and Abnormal Developing Human Forebrain

**DOI:** 10.3389/fnana.2017.00112

**Published:** 2017-12-04

**Authors:** Karen B. Larsen

**Affiliations:** ^1^Department of Pathology, Rigshospitalet, University Hospital of Copenhagen, Copenhagen, Denmark; ^2^Department of Neuropathology and Ocular Pathology, John Radcliffe Hospital, Oxford University Hospital, Oxford, United Kingdom

**Keywords:** fetal, stereology, neurons, glial cells, IUGR, down syndrome

## Abstract

Human fetal brain development is a complex process which is vulnerable to disruption at many stages. Although histogenesis is well-documented, only a few studies have quantified cell numbers across normal human fetal brain growth. Due to the present lack of normative data it is difficult to gauge abnormal development. Furthermore, many studies of brain cell numbers have employed biased counting methods, whereas innovations in stereology during the past 20–30 years enable reliable and efficient estimates of cell numbers. However, estimates of cell volumes and densities in fetal brain samples are unreliable due to unpredictable shrinking artifacts, and the fragility of the fetal brain requires particular care in handling and processing. The optical fractionator design offers a direct and robust estimate of total cell numbers in the fetal brain with a minimum of handling of the tissue. Bearing this in mind, we have used the optical fractionator to quantify the growth of total cell numbers as a function of fetal age. We discovered a two-phased development in total cell numbers in the human fetal forebrain consisting of an initial steep rise in total cell numbers between 13 and 20 weeks of gestation, followed by a slower linear phase extending from mid-gestation to 40 weeks of gestation. Furthermore, we have demonstrated a reduced total cell number in the forebrain in fetuses with Down syndome at midgestation and in intrauterine growth-restricted fetuses during the third trimester.

## Introduction

The neocortex is the main locus of cognition in the human brain and its expansion relative to that in non-human primates is a main anatomic distinction. Since the number of neocortical neurons is generally accepted to bear some relation with cognitive abilities, several stereological studies have quantified neocortical cell numbers in brains of adult humans and various species (Korbo et al., 1990; Pakkenberg and Gundersen, 1997; Jelsing et al., 2006; Christensen et al., 2007; Eriksen and Pakkenberg, 2007; Walløe et al., 2010; Fabricius et al., 2013). On the other hand, there have been few developmental studies of cell numbers in the human fetal and neonate forebrain (Winick, 1968; Dobbing and Sands, 1973; Rabinowicz et al., 1996), none of which employed stereological methods. This paucity may well reflect technical and ethical issues arising in studies of the fetal human brain. Furthermore, many earlier studies of brain cell numbers have been based upon assumptions that all cells (counting objects) are of uniform shape and size or are isotropically orientated in the specimen (Weibel and Gomez, 1962; Rose and Rohrlich, 1987). Violation of these assumptions give rise to significant bias in the estimation of cell numbers, but stereological methods accommodate these factors. Stereology is an application of spatial sampling that enables researchers to obtain three-dimensional information about the distribution of objects observed in a slice or section. In general, it is not possible to determine the exact cell numbers in large structures, but estimation of total cell numbers through application of stereological principles is efficient, i.e., “with a low variability after spending a moderate amount of time,” and unbiased, that is to say “without systematic deviation from the true value” (Gundersen et al., 1988a,b). Thus, the stereological methods yield reliable and efficient estimates of total cell numbers even in structures as complex and heterogenous as the human fetal brain. In order to support statistical comparison between groups, the stereological sampling frequency can be adjusted to give optimal precision without unwarranted effort (Gundersen et al., 1988a,b; West, 2012).

A series of stereological studies has contributed invaluable knowledge of the proliferation of total neocortical cell numbers in human fetal brain by use of the optical fractionator (Samuelsen et al., 2003, 2007; Larsen et al., 2006, 2008, 2010). The optical fractionator design is an ideal method to obtain total cell numbers in the fragile fetal brain due to the minimum of handling of the tissue. There follows a detailed and critical account of how to apply the optical fractionator scheme for robust estimation of cell numbers in the human fetal forebrain across normal and abnormal intrauterine development. Furthermore, there are some comments upon already published results. Since our histological methods did not distinguish between neurons and glial cells, the total cell numbers is a sum of future neurons and glial cells.

## Development of the human fetal forebrain brain and its transient fetal zones

Main events in the development of the human brain include neuronal proliferation, migration, differentiation, synaptogenesis, pruning and myelination. Deviations from the normal developmental sequence due to genetic or environmental factors give rise to cerebral malformation. Severe misdevelopment of the brain can be diagnosed by post mortem examination or through macroscopic imaging techniques *in utero*. However, detecting subtle changes in cell numbers requires quantitative measurements by microscopy. We have focused our investigations on the total cell numbers in the maturing neocortex or in transient fetal zones destined to develop into neocortex (Kostovic and Judas, 1995). More than 40 years ago, the Boulder Committee, founded by the American Association of Anatomists, described and named these zones, which have no direct counterpart in the adult brain (The Boulder Committee, 1970). This description was revised by Bystron et al. (2008).

During human fetal weeks 3 and 4, the telencephalic wall contains only *the ventricular zone* (VZ). Neuroepithelial cells then constitute a homogenous cell population in the neural tube, and the major histogenic event is intensive symmetric proliferation where single progenitor cell first give rise to two progenitor cells before the onset of neurogenesis (Kornack, 2000). The first phase of neurogenesis starts at approximately fetal week 5, which occurs in conjunction with differentiation of the primordial neuroepithelial cells into radial progenitor cells. They provide a scaffold for migration of newborn cells to their predetermined position, while also acting as progenitor cells for more neurogensis (Malatesta et al., 2000; Miyata et al., 2001; Noctor et al., 2001; Heins et al., 2002). At fetal week 6, *the subventricular zone* (SVZ) arises on the border of the VZ through accumulation of outer radial glial cells and intermediate progenitors lacking an attachment to the ventricular surface. Even through early human postnatal life, immature neurons continue migrating from the SVZ to specific targets such as the olfactory bulb and prefrontal cortex (Sanai et al., 2011; Khodosevich et al., 2013; Wang et al., 2013; Paredes et al., 2016), but this process declines in early childhood (Sanai et al., 2011; Paredes et al., 2016).

The intensive proliferation in the SVZ leads to formation of the ganglionic eminence in the basoventral part of the telencephalon. The ganglionic eminence later develops into the basal ganglia, but is in rodent also the source of neocortical inhibitory GABAergic interneurons, which migrate dorsally into the neocortex in a process known as tangential migration (Anderson et al., 1997; Tamamaki et al., 1997). Human neocortical interneurons are also derived from the ganglionic eminence (Letinic et al., 2002; Fertuzinhos et al., 2009), but the majority of neocortical interneurons arise in the VZ and SVZ of the dorsal telencephalon itself (Letinic et al., 2002).

The Boulder Committee originally suggested that before any post mitotic cells appear in the prospective cortex, the subpial processes of ventricular cells constitute a cell sparse layer which they termed *the marginal zone* (MZ). Increasing evidence has revealed that no such subpial layer exists prior to the tangential invasion of the first cortical neurons (Bystron et al., 2005, 2006), such that the term MZ is more properly employed after the formation of the cortical plate (Uylings, 2000; Bystron et al., 2008). A compartment consisting of heterogeneous post migratory cells and neuropil lying between the proliferative zones and the pial surface of the dorsal telencephalon before the appearance of the cortical plate is known as the preplate.

During fetal weeks 8 and 9, the first migratory wave of cells form *the cortical plate* (CP), a distinctive layer of tightly packed neurons. The CP divides the preplate into the MZ and the subplate/intermediate zone. *The intermediate zone* (IZ), which contains both radially and tangentially migrating cells together with axons, constitutes the future white matter. *The subplate zone* (SP) transiently appears between the IZ and the CP. The first phase of synaptogenesis is thought to occur in the SP, which is also a target for afferents arising from the brain stem, basal forebrain, thalamus and the ipsi- and contralateral cortices (Kostovic and Rakic, 1990). Together with the neighboring CP and the MZ, the SP will develop into the future cortical gray matter. The SP undergoes intensive reorganization and reaches its maximum thickness at 22 fetal weeks, begins to regress after fetal week 35, and ultimately disappears by the second postnatal year (Kostović et al., 2014).

## Methods

### Optical fractionator

The optical fractionator combines two stereological tools: the optical disector and the fractionator sampling design (Gundersen, 1986; Gundersen et al., 1988a,b; West et al., 1991). A unique aspect of the optical disector method lies in the lack of any assumptions about size, shape, orientation or distribution of the particles (i.e., cells) to be counted, and that it samples with a probability that is proportional only to their number (Gundersen, 1986). The optical disector in which the cells are counted directly is an unbiased three-dimensional probe with a well-defined volume. In particular, the fractionator is a systematic uniform random sampling (SURS) scheme, which assures that the fraction of the structure of interest is known. The basic principle of the fractionator is quite simple: one investigates a known fraction of the whole structure of interest, for example 1:200, and then counts every cell in that fraction. The total number of cells in the entire structure is the number counted in that fraction multiplied by the inverse sampling fraction, in this case 200.

The optical fractionator entails counting within optical disectors using SURS that constitute a known fraction of the structure to be analyzed. This fraction is obtained by sampling a known fraction of the section thickness, under a known fraction of the area encompassing the structure of interest, and for a known fraction of the total number of sections comprising the structure of interest. The method requires that the whole structure be fully available, and that the structure can be exhaustively cut into sections of a thickness exceeding the height of the dissector and guard zones (West et al., 1991). For unbiased estimation, the sampling design must be based on systematic, uniform, and random sampling with a predetermined periodicity from a random starting position within the first interval of the sectioning (Gundersen and Jensen, 1987). Such a sampling ensures that every part of the structure has a priori the same chance of being included in the final sample. Then an estimate of total cell numbers is obtained, but the volume of the region of interest is not known. The method is insensitive to tissue changes due to fixation or preparation, and neither the volume of the region of interest, the cell density, nor the magnification need be known.

These properties make the optical fractionator design an ideal procedure to estimate total cell numbers in fragile and distorted tissue such as fetal brain. Because fetal brain has low myelin content and high water content, tissue handling and processing almost always results in some fragmentation. Therefore, handling of fetal brain prior to paraffin embedding must be minimized. By using the optical fractionator method, the only handling before paraffin embedding is cutting the chosen hemisphere into either two or three blocks before further treatment, or simply directly embed the chosen hemisphere in paraffin depending upon the size of the brain. Thus, the sampling process begins only after cutting the sections.

### Practical application of the optical fractionator to the fetal brain

**Figure 1:**
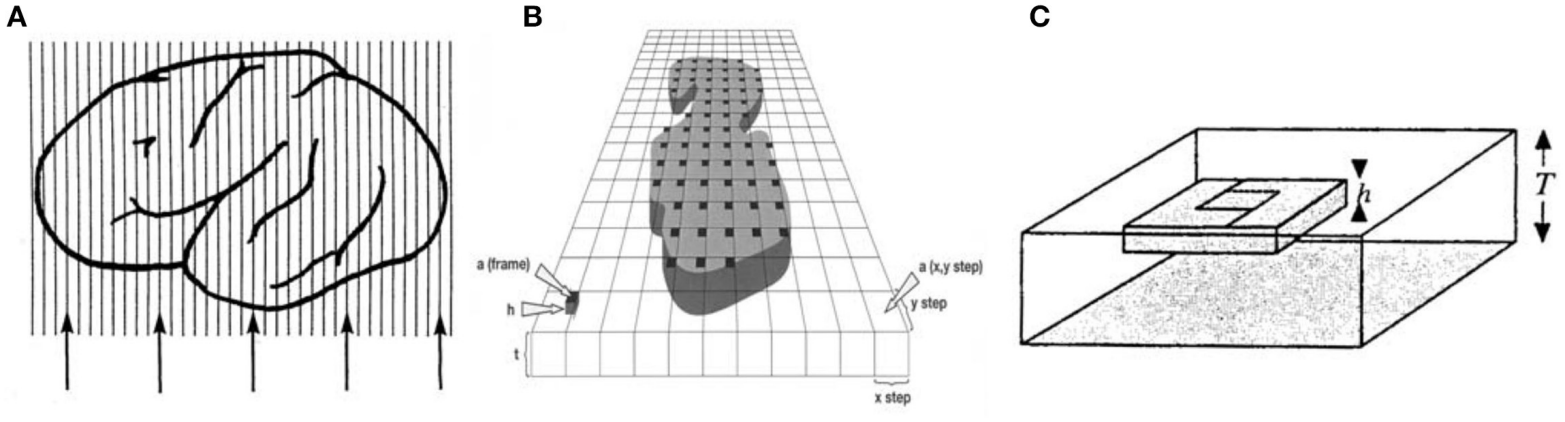
**(A)** A known fraction, the section sampling fraction, *ssf*, is chosen. In this case five sections (lines are representing sections) are chosen from the object of interest, and **(B)** a step length = (x, y) and a counting frame area = a(frame) are applied. After placement of a 2D unbiased counting frame, a(frame) in the first predetermined x,y interval, the remainder of the positions of the counting frame in the x,y intervals are repositioned using a stepping motor. **(C)** Each black square is an optical disector with a fixed height, *h*. Modified from Figure 5.19 in Howard and Reed (2010).

**Figure 2:**
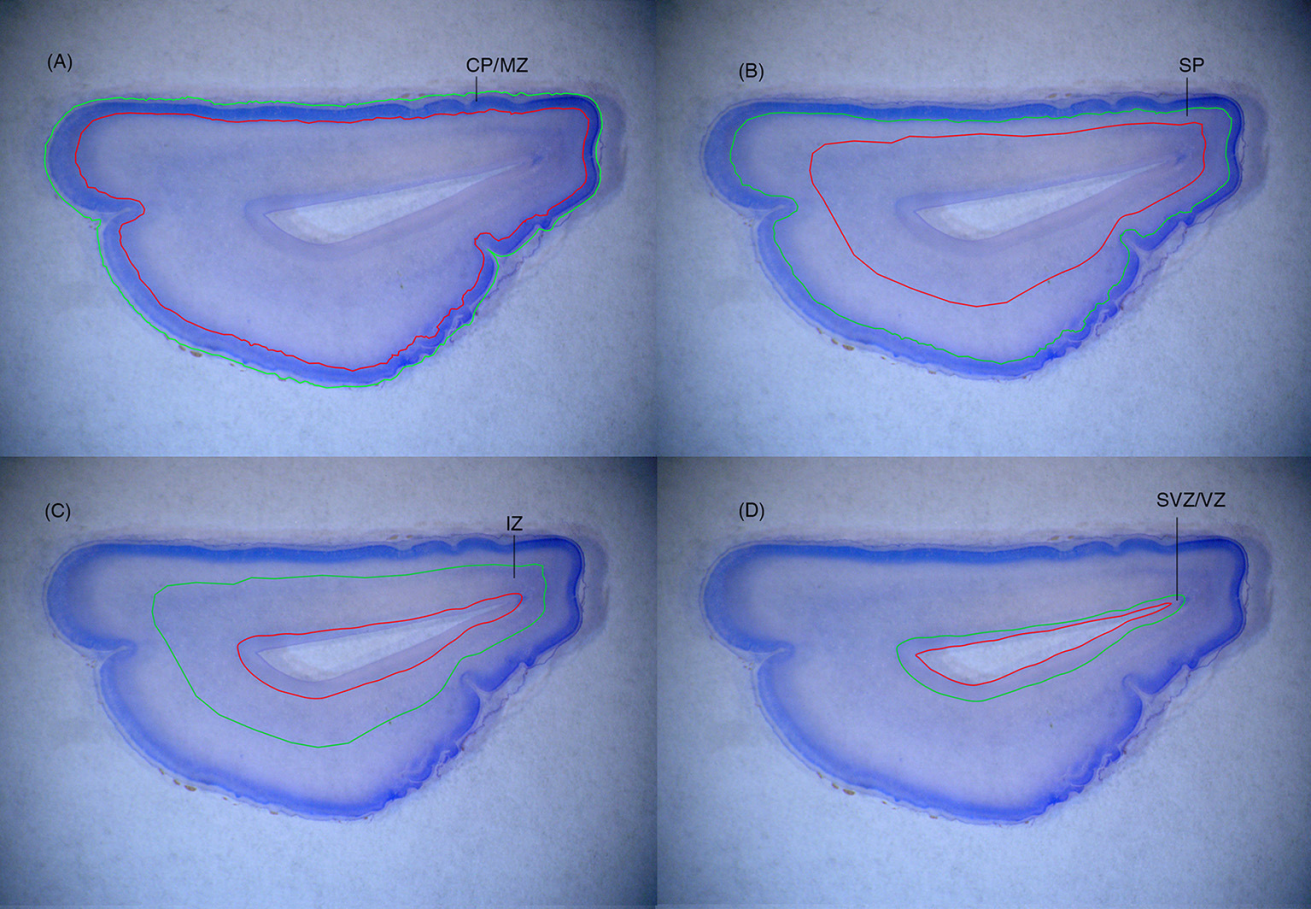
**(A)** The inclusion line (green line) for the CP/MZ is drawn according to the outer pial surface, whereas the exclusion line (red line) for the CP/MZ is made at the interface between the CP/MZ and the SP. **(B)** After the cells in the CP/MZ are counted within optical disectors for this zone, the former exclusion line of the CP/MZ becomes the inclusion line for the underlying SP and a new exclusion line is drawn at the interface between the SP and the IZ. **(C,D)** The same procedure is applied to the IZ and the SVZ/VZ, thus ensuring that no areas (and thus no cells) are omitted or counted twice due to inconsistent delineation.

**Figure 3:**
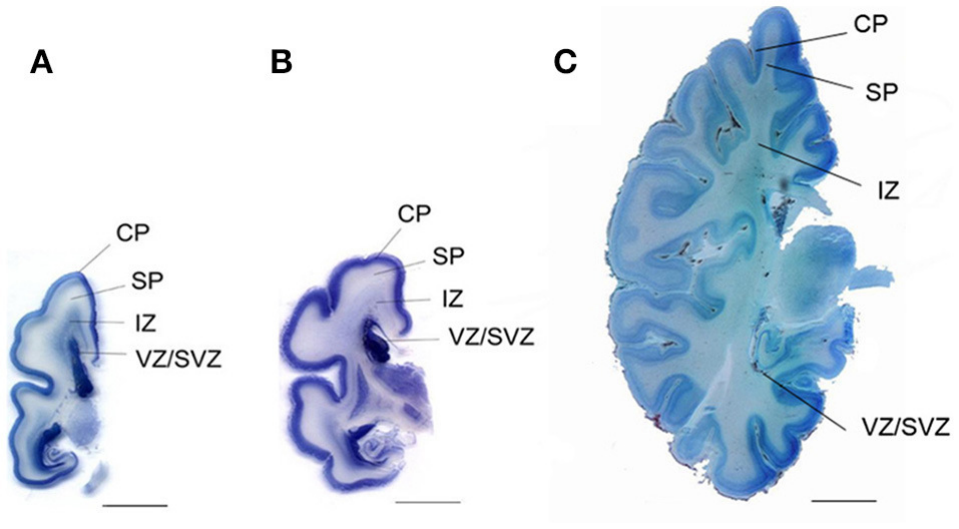
“Coronal sections from a 24-weeks **(A)**, a 25-weeks **(B)**, and a 40-week-old **(C)** human fetus at the level of the basal ganglia. CP, cortical plate; SP, subplate; IZ, intermediatezone; VZ/SVZ, ventricular zone/subventricularzone. Scalebar = 1 cm.” Reproduced with permission from Walløe et al. (2014).

**Figure 4:**
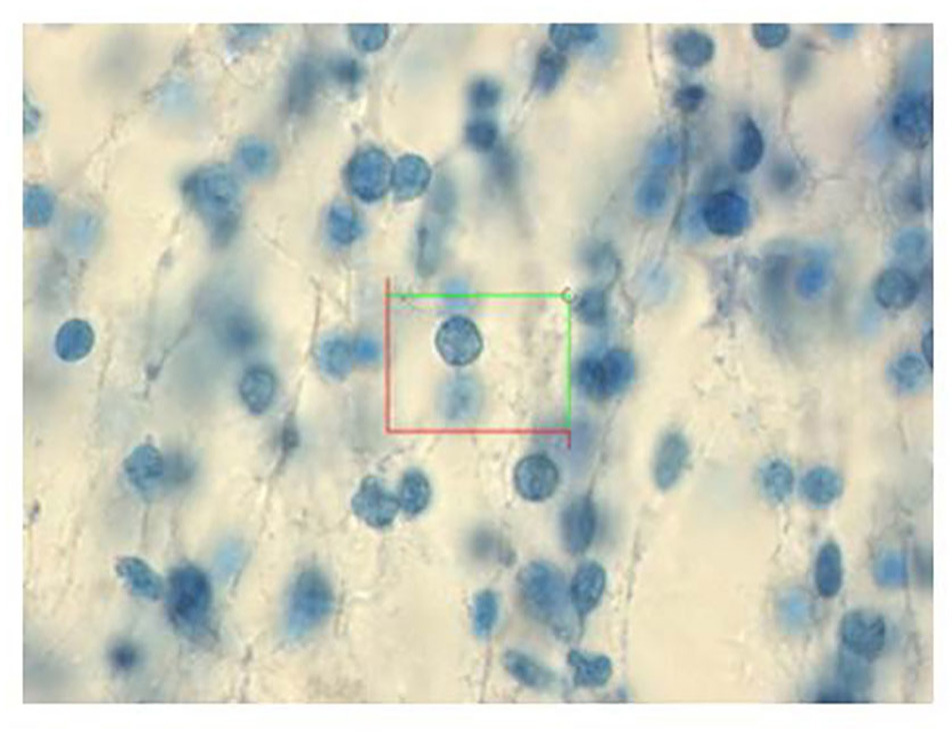
“An optical disector in the neocortex of a 19-week fetus. The cells were counted when the nucleus came into focus in the optical disector. Paraffin, Giemsa—stained section, 40 mm thick, magnification 3100x.” Reproduced with permission from Larsen et al. (2008).

Application of the optical fractionator for fetal tissue entails the following steps:

1. Since the fetal brain is soft and vulnerable, the 10% formalin solution used for postnatal brain is unsuitable. Instead, we harden the tissue by fixation in 25% saturated picric acid/20% formalin for 4 weeks prior to cutting. The left or right hemisphere is chosen systematically at random.
2. Hemispheres are cut into either two or three blocks before further treatment, or are directly embedded in paraffin depending on the size of the brain. Primary blocks are then sectioned coronally on a sledge microtome at 40 μm proceeding from the frontal to the occipital pole. Every sampled section is then mounted on glass slides coated with gelatine-chromepotassium sulfate dodecahydrate and immediately thereafter dried at 40°C for 24 h. Before staining, the section are heated to 60°C for 30 min, hydrated in xylene for 2 × 25 min in a 50:50 mixture of xylene and acetone dimethyl acetyl hydrochloride containing 1 μl hydrochloric acid per ml acetone dimethyl acetate, and then immersed for 2 × 30 min in the acetone dimethyl acetal hydrochloride, followed by washing for 5 min in distilled water.
3. The sections are stained by immersion for 45 min in a modified Giemsa stain containing: 50 ml Giemsa stain stock solution and 200 ml KHPO_4_, 67 mmol/L, pH 4.5, the mixture being filtered just before use. Then the sections are differentiated and dehydrated by passage through 0.5% acetic acid in 96% alcohol for 1 to 5 min, 5 min in 99% alcohol and 10 to15 min in xylene.
4. Beginning from the frontal pole, sections selected according to a predetermined periodicity are sampled after a random start within the first sampling period. A known fraction, the section sampling fraction, *ssf*, is chosen aiming to achieve about 10 sections per brain. If *ssf* were chosen to be 1/100th, a random start within the first predetermined period could be, for example, section 20, and the next would then be section 120 (Figure 1A).
5. For each sampled section, the different zones in the neocortex are delineated (Figure 2) by making a fiducial mark in Indian ink “corresponding to the borders histologically verified using a stereo microscope. The neocortex develops a prominent CP and SP, while the archicortex develops an exceptionally wide MZ and a thin convoluted CP without a real SP. Furthermore, the telencephalic wall is slightly curved in this area. The paleocortex never develops a true CP” (Kostovic and Judas, 1995; Samuelsen et al., 2003). It should be noted that gyration patterns and sizes of fetal zones change dramatically with increasing fetal age (Figure 3).
6. The borders of the fetal zones are then transferred to the Computer Assisted Stereological Test Grid System (CAST grid system, Olympus Denmark). The total number of cells is estimated separately in each fetal zone, as in step 7 below. The inclusion line for the CP/MZ is drawn according to the outer pial surface, whereas the exclusion line for the CP/MZ is made at the interface between the CP/MZ and the SP. After the cells in the CP/MZ are counted within optical disectors for this zone, the former exclusion line of the CP/MZ becomes the inclusion line for the underlying SP and a new exclusion line is drawn at the interface between the SP and the IZ. The same procedure is applied to the IZ and the SVZ/VZ, thus ensuring that no areas (and thus no cells) are omitted or counted twice due to inconsistent delineation (Figure 2).
7. A computer monitor displays the microscopic view obtained with a 100x oil objective of high numerical aperture. The step length (*x,y* interval) and the size of the counting frame *a*(frame) of the optical disector must be designed to achieve counting of ~100–200 cells in each fetal zone. Furthermore, for correct counting the area of the counting frame of the optical disector should be set so as to count 2–4 cells per frame. Cells are counted when the nucleus come into focus (Figure 4). After random placement of a two-dimensional unbiased counting frame of the optical disector in the first predetermined *x,y* interval, the remaining counting frame positions of the optical disector are displaced in the *x,y* intervals using a stepping motor. The area sampling fraction, *asf*, is then calculated as the ratio between the area of the counting frame, *a*(frame) and the area associated with each displacement in x and y, *a*(*x,y* interval). Thus, *asf* = *a*(frame)/*a(x,y* interval) (Figure 1B).
8. The mean thickness, *t*_*q*_ (the number-weighted mean section thicknesss), should be calculated from measurements made at defined intervals such as every 5th optical disector. This is accomplished by locating the surface of the section and registering 0 in the z-axis when the first feature appears in focus, and then moving to the bottom of the section where features are out of focus, and noting the thickness of the section. The fixed height of the optical disector must be less than the total thickness to avoid artifacts at the top and bottom surfaces of sections (these volumes are known as the guard zones). Since the cells are only counted in *h*, the height sampling fraction, *hsf*, is calculated as:
  $$\begin{array}{l} hsf=\frac{h}{{\bar{t}}_{Q^{-}}}\text{ for }{\bar{t}}_{Q^{-}}=\frac{\underset{i}{\sum }\left(t_{i}q_{i}^{-}\right)}{\underset{i}{\sum }q_{i}^{-}} \end{array}$$
  where t_i_ is the local section thickness centrally in the i*th* counting frame with a disector cell count of $q_{i}^{-}$ (Dorph-Petersen et al., 2001). A Heidenhain microcator measures movement in the z-axis with a precision of 0.5 μm (Figure 1C).
9. For one brain hemisphere, the cells counted in a fetal zone is denoted as ΣQ^−^. Then the total bilateral number of cells in that particular fetal zone is estimeted as:
  $$\begin{array}{l} N:=\frac{1}{ssf}x\frac{1}{asf}x\frac{1}{hsf}x\sum Q^{-}x2 \end{array}$$
  The doubling to provide a bilateral estimate is allowed because the hemisphere has been selected according to random choice. The total number of cells in the neocortical part of the telencephalic wall is obtained by summing N of each fetal zone in that specimen.
10. Before committing to the use of valuable samples, it is recommended to perform a pilot study to address the various technical issues raised in the above, aiming to obtain an ideal and efficient sampling design. The final design will, for example, depend upon the extent of shrinkage and if there is an either homogeneous or heterogeneous distribution of the cells in the structure of interest. Furthermore, it is of great importance to calibrate the microscope stage so that the commanded step lengths are correctly executed and to check the staining throughout the tissue.

### Precision of the estimate CE using the optical fractionator

In addition to the practical application of the optical fractionator to cell counting in human fetal brain, certain statistical considerations must be addressed to obtain accurate estimates of cell numbers. The total observed variance (CV^2^) is the sum of the biological variance and the mean sampling variance, where CV can be calculated as the standard deviation of the estimate divided by the group mean. The coefficient of error, CE = SEM/mean, which indicates the precision of the estimate for a single brain, depends upon the sampling design, and describes both the variance between sections and the variance within sections from a single brain. In essence, the CE describes the quality of the measurement, which refers to the difference between the estimate and the true value. We estimate CE as follows:

$$\begin{array}{l} \text{CE}=\frac{\sqrt{Var_{surs}+Noise}}{\sum Q^{-}}, \end{array}$$

Here, noise is the sum of counted particles and Var_SURS_ is the estimated variance according to Systematic Uniform Random Sampling (SURS) (Gundersen and Jensen, 1987; Gundersen et al., 1999). The SURS takes into account the systematic nature of the sampling, and this is superior to and more efficient than independent sampling. The Var_SURS_(*N*) is obtained from the formula:

$$\begin{array}{l} \text{Va}{\text{r}}_{\text{SURS}}\left(N\right)=\frac{\left(3\left(A-Noise\right)-4B+C\right)}{240}, \end{array}$$

where the systematic section series of the particle count is denoted *f*_1_*, f*_2_ …… *f*_*n*_ and.

$$\begin{array}{l} \text{A}=\overset{n}{\underset{i=1}{\sum }}{fi}^{2};\text{B}=\overset{n-1}{\underset{i=1}{\sum }}fifi+1\text{ and C}=\overset{n-2}{\underset{i=1}{\sum }}fifi+2 \end{array}$$

These CE calculations are valid when samples are not independent and are systematically chosen in a uniform and random way. The nature of the variation between samples in our studies are believed to be of the smoothness class *m* = 1, as we did not observe any abrupt variation between samples. Thus, the numerator in the calculation of Var_SURS_ should be divided by 240 and not 12 (Gundersen et al., 1999).

The biological variance can be calculated when CV and CE are known. The researcher cannot control the biological variance CV_biol_, which arises from actual differences between individuals, but can determine the mean sampling variance (CE). It is obvious that if the biological variation in cell numbers is high, there is little effect on the total observed variance from adjusting the sampling design whereas the sampling frequency should be increased if the sampling variation is the main contribution to the total observed variance of the estimate.

## Application of the optical fractionator to the fetal and neonatal brain in order to estimate total cell numbers in the normal and abnormal fetal and neonatal human forebrain

**Figure 5:**
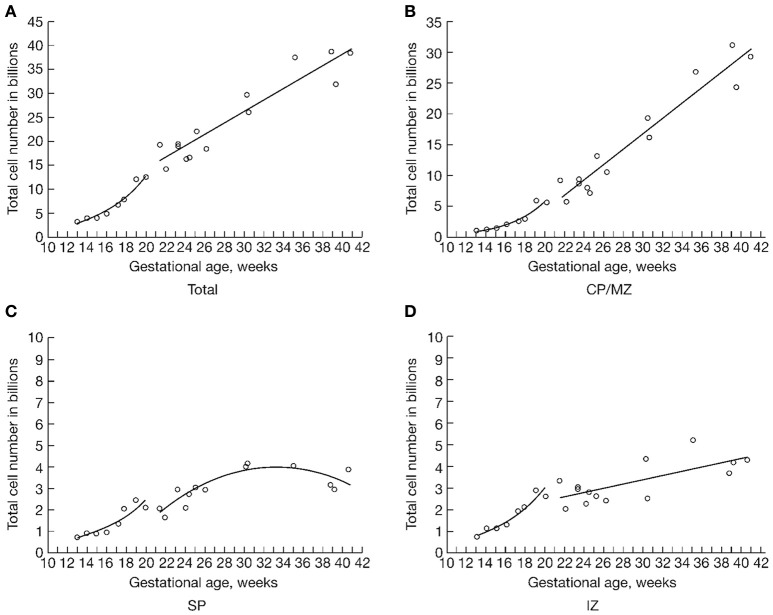
“The total number of cells in billions (10^9^) in three major developmental zones of the human fetal forebrain **(B,C,D)** (CP/MZ, SP, IZ) and in the summation of all four zones **(A)** (CP/MZ, SP, IZ, VZ/SVZ), representing the total number of cells in the entire human fetal forebrain. The material has been allocated into two study periods: one from 13 to 20 weeks of gestation and another from 22 weeks of gestation to term. Due to the limited number of cases during the period from 18 to 22 weeks of gestation, we could not determine which of the two mathematical models that gave the best fit in that transitional interval.” Reproduced with permission from Samuelsen et al. (2003).

Many recent studies have investigated the molecular background of neurogenesis, patterning of brain regions, and circuit formation in the developing human brain. However, very little has been known about the prenatal development of the human fetal brain in terms of growth in cell numbers. The first stereological study applying the optical fractionator to cell counting in human fetal brain appeared in 2003 (Samuelsen et al., 2003). In that study we estimated total cell numbers in selected zones of the fetal neocortical cerebral wall in 22 human fetuses aged 13 to 41 gestational weeks. Since our histological methods do not distinguish between neurons and glial cells, we report total cell numbers, i.e. the sum of future neurons and glial cells. We found that the growth in cell numbers in the human fetal forebrain appears to be two-phased, with an initial exponential phase from 13 to 20 gestational weeks, followed by linear phase from 22 weeks of gestation until term. From 13 to 20 weeks of gestation the total number of cells increased four-fold, from 3 × 10^9^ to 13 × 10^9^ cells, and increased by a further factor of 3 to 38 × 10^9^ cells at term (Figure 5). During the second half of gestation, we estimated 170 million new brain cells per day in the entire neocortical wall, equivalent to 2,000 new cells each second.

We also estimated the total number of cells in the developing ganglionic eminence in 13 human fetuses aged 22 to 29 fetal weeks with use of the optical fractionator (Larsen, 2010; Larsen et al., 2010). From 11 to 20 fetal weeks, the total number of cells in the ganglionic eminence increased six-fold from 0.47 × 10^6^ to 2.86 × 10^6^, whereafter total cell numbers declined until the structure finally disappeared around term. In that study, we demonstrated that the ganglionic eminence, like the neocortex, exhibits an exponential development of total cell numbers from 10 to 20 fetal weeks. Our stereological data are in agreement with the volumetric data from Huang et al. (2009), who used diffusion tensor imaging of post mortem human fetal brains to chart the increasing volume of the ganglionic eminence during the second trimester. Previous studies have identified the ganglionic eminence as an important source of neocortical inhibitory interneurons and oligodendrocyte progenitor cells (Anderson et al., 1997; Tamamaki et al., 1997; Letinic et al., 2002; Rakic and Zecevic, 2003; Ma et al., 2013), which may well drive the exponential development in total cell numbers lasting until mid-gestation. Although the ganglionic eminence disappears around birth, the production of neocortical inhibitory interneurons continues into early postnatal life (Arshad et al., 2016), and remnants of the SVZ produce cells until early infancy (Del Bigio, 2011).

In the final study to investigate the normal developing brain in term infants, we estimated total cell numbers in 10 normal neonate brains within the CP/MZ, the SP, the IZ and the SVZ/VZ (Larsen et al., 2006) by applying the optical fractionator. The gestational ages ranged from 38 to 42 weeks. In that study, the total number of cells in the entire neocortical part was almost 33 × 10^9^ in the human neonate forebrain, and around 24 × 10^9^ in the CP/MZ. Other studies of the adult neocortex indicate a total of 50 × 10^9^ cells in females and 65 × 10^9^ in males (Pelvig et al., 2008), of which 19 × 10^9^ are neurons in females and 23 × 10^9^ are neurons in males (Pakkenberg and Gundersen, 1997). Compiling these studies indicates that the total number of neurons in neonates equals the total number in adults, whereas glial cells continue to proliferate in postnatal development. Indeed, the first stereological study of total cell numbers during neocortical development of rat pups found similar growth trajectories of neurons and glial cells (Behnam-Rassoli et al., 1991). In agreement with these findings, two recent stereological studies (Kjær et al., 2016; Sigaard et al., 2016) showed linear increases in both oligodendrocyte and astrocyte numbers during the first 3 years of human life in humans, and that the number of neocortical neurons has already attained the adult population at least at term.

The growth in fetal brain cell numbers is so complex and rapid that any disruption is apt to derail the normal growth program away from delicately predetermined interrelationships, likely resulting in anatomic and functional deficits persisting to maturation. With this in mind, we have investigated total cell numbers in the forebrain in fetuses with Down syndrome (DS) and in intrauterine growth-restricted (IUGR) fetuses, which constitute relative common conditions of abnormal fetal development. Alterations of brain development and intellectual disabilities occur in DS (Schmidt-Sidor et al., 1990; Devenny et al., 1996, 2000; Krinsky-McHale et al., 2002; Nelson et al., 2005). We used the optical fractionator to compare the total cell numbers in forebrain of 4 DS fetuses aged 19 weeks of gestation to 8 normal control fetuses The total cell number was 34% lower in the neocortical part of the cerebral wall of DS fetuses (6.85 × 10^9^) compared to normal controls (10.4 × 10^9^). We suppose that the intellectual disability often occurring in DS may arise from a structural deficit in the human fetal brain already present in the second trimester (Larsen et al., 2008).

We also used the optical fractionator to to estimate the cell numbers in the forbrain of nine severely affected IUGR fetuses and 15 control fetuses with gestational ages ranging from 19 to 41 weeks. The total cell number in the CP/MZ was significantly lower in IUGR fetuses compared to controls, and the daily increase rate in the CP/MZ of IUGR fetuses was only half of the controls; whereas control fetuses acquired an average of 173 million CP/MZ cells per day from midgestation to term, the IUGR fetuses acquire only 86 million new cells per day (Samuelsen et al., 2007).

## Methological considerations using the optical fractionator

A large number of stereological studies have used the stereological ratio estimators (density multiplied by reference volume) to quantify total cell numbers in the human brain (Pakkenberg and Gundersen, 1997; Andersen et al., 2003; Pelvig et al., 2008; Karlsen and Pakkenberg, 2011; Karlsen et al., 2014). In that approach, the number of cells is counted in optical disectors, knowing the dimensions of the disector and the total volume (see e.g., Pakkenberg and Gundersen, 1997). The method of multiplying density by the reference volume is obviously a good choice when volume and total cell numbers are both matters of interest. It is then possible to obtain reliable mean estimates of both parameters for making comparisons by group or treatment. The adult brain suffers less unpredictable dimensional change during tissue processing than typically occurs in fetal material.

Due to the unpredictable shrinkage and distoration during fixation of fetal brain, it is of questionable value to attempt an estimation of volumes and densities. However, the optical fractionator method for estimating total cell numbers is the obvious choice for fetal brain studies, because results are robust to deformation, shrinkage, and swelling of the reference space. Furthermore, it is easier to apply the optical fractionator, because no reference volume estimate is required.

Despite the advantages of the optical fractionator method, some pitfalls should be considered.

- Since only total cell number estimates are obtained with the optical fractionator method, it is sensitive to any errors occuring during the sampling procedure. One such error could be due to incorrect calibration of the microscope stage, resulting in unreliable step lengths. Since the optical fractionator gives only total cell number, neither density nor volume estimates will help to reveal a calibration error.
- If there are many artifacts in the tissue, it is necessary to keep track of the fraction of disectors that are uncountable, so this fraction can be properly included in the final estimation of total numbers.
- Cells in the SVZ/VZ lie very closely together and have a tendency to cluster, intermixed with acellular areas. In such circumstances of heterogeneity of cell distribution, it is important to use a sampling design with relatively small counting frames and small step lengths in order to obtain robust and precise estimates of total cell numbers.
- Often, relatively few (8–12) sections suffice when using the optical fractionator. This is due to low variation in count between sections during the calculation of the CE, since the counts are systematically related. Thus, the numerator can be divided by 240 instead of 12 in the Var_SURS_ (N) equation, see section Precision of the Estimate CE using the Optical Fractionator. The sampling scheme must be strictly systematic if low sampling is to yield the desired amount of precision in the estimate of total cell numbers (West et al., 1991). However, if cell counts between neighboring sections vary considerably, a greater number of sections must be sampled to obtain an acceptable precision.
- When applying the optical disector, it is necessary to introduce guard zones, as noted above. This is due to optical limitations, and also to avoid bias from lost tissue fragments at the cut surface of sections. While especially important in fragile fetal tissue, the use of guard zones can bias total cell number estimates under certain circumstances. This is because the disector placement is not uniformly random within the section thickness, as can happen if there is a heterogeneous cell distribution along the z-axis. In sections thinner than 20 μm, the relative error in measuring the section thickness increases dramatically, so thicker sections are to be preferred. Also, it is recommended to use microscope objectives with high numerical objectives to provide a very shallow depth of focus in the images (West et al., 1991).

If these recommendations and stipulations are satisfied, total cell number estimates with the optical fractionator are indeed “unbiased for all practical purposes” (Gundersen et al., 1988a,b). Correct use of the optical fractionator requires some expertise with stereological tools, and therefore might not be the preferred method for researchers with no access to expert guidance. In such circumstances, multiplying the density by the reference volume method might be more appropriate, as it is more robust and less vulnerable to sampling design. In the present context, there is a particular need to minimize handling of fragile fetal tissue and when all requirements are observed, the optical fractionator method is the perfect choice to estimate total cell numbers in fetal brain.

## Conclusion

In the three decades since the landmark study showing how to obtain unbiased estimation of individual particle numbers was published (Sterio, 1984), the optical disector method has matured to a general tool for quantifying the number of cells in brain and other organs. Subsequently, the optical fractionator method has made it possible to overcome important methodological problems arising from unpredictable shrinkage during fixation of the fetal brain and furthermore, several user-friendly software programs exist which can assist the un-experienced researcher with the calculations and evaluation of the estimate quality.

Despite the widespread knowledge of the advantages of unbiased stereological methods, many quantitative studies continue to use cell densities in sections. However, the volume/reference trap should be avoided. When using the density multiplied by reference volume, the reference volume and the numerical cell density both contribute to the final readout of total cell counts. To avoid bias, stereologists should always report the numerator and denominator instead of simply reporting cell density. It is better to graph the explicit number or length as a function of the volume. Density, a ratio between two variables, is of limited value unless used to obtain total cell numbers. Alternatively, one may use the optical fractionator to obtain directly the total cell numbers.

Present histological methods do not distinguish between neurons and glial cells until term. Thus, we estimate total cell numbers and net proliferation, although certain transient features can be seen to decline in size and cell counts during late gestation. In future studies the use of an apoptotic marker in combination with stereological methods in the fetal brain could contribute to a more exact knowledge of the growth in cell numbers. Furthermore, other cell-labeling techniques could eventually give separate estimates of neurons and glial cells during human fetal brain development.

In summary, we have applied the optical fractionator to investigate total cell numbers of the developing human fetal and neonate forebrain. Remarkably, the production of billions of cells, many of which are neurons, occurs during a rather brief span of intense and exponential proliferation. Furthermore, we have demonstrated a reduced total cell number in the forebrain in DS fetuses at midgestation and in IUGR fetuses during the third trimester.

## Author contributions

The author confirms being the sole contributor of this work and approved it for publication.

### Conflict of interest statement

The author declares that the research was conducted in the absence of any commercial or financial relationships that could be construed as a potential conflict of interest.
